# Feasibility and acceptability of a remotely delivered, home-based, pragmatic resistance ‘exercise snacking’ intervention in community-dwelling older adults: a pilot randomised controlled trial

**DOI:** 10.1186/s12877-022-03207-z

**Published:** 2022-06-25

**Authors:** Jackson J. Fyfe, Jack Dalla Via, Paul Jansons, David Scott, Robin M. Daly

**Affiliations:** 1 Institute for Physical Activity and Nutrition (IPAN), School of Exercise and Nutrition Sciences, Deakin University, Geelong, Australia; 2 Nutrition and Health Innovation Research Institute, School of Medical and Health Sciences, Edith Cowan University, Perth, Australia; 3 Department of Medicine, School of Clinical Sciences at Monash Health, Monash University, Clayton, Australia

**Keywords:** Resistance training, Home exercise, Muscle strength, Physical function, Older adults

## Abstract

**Background:**

Very few older adults meet current muscle strengthening exercise guidelines, and several barriers exist to supervised, community-based resistance exercise programs. Older adults therefore require access to feasible resistance exercise modalities that may be performed remotely. This pilot study assessed the feasibility and acceptability of undertaking a four-week home-based resistance ‘exercise snacking’ intervention (performed either once, twice, or thrice daily) when delivered and monitored remotely in older adults.

**Methods:**

Thirty-eight community-dwelling older adults [mean ± SD age 69.8 ± 3.8 y, 63% female] were randomised to complete resistance ‘exercise snacks’ (9-minute sessions) either once (*n* = 9), twice (*n* = 10), or thrice (*n* = 9) daily, or allocated to usual-activity control (n = 10). Exercise adherence and adverse events were assessed using an exercise diary, and acceptability of the intervention was explored using an online questionnaire. Physical function [balance, 5-times sit-to-stand (STS), and 30-second STS tests] was assessed remotely at baseline and follow-up using videoconferencing.

**Results:**

The intervention was feasible and safe, with 100% participant retention, high adherence (97, 82, and 81% for once, twice, and thrice daily, respectively), and only two adverse events from a total of 1317 ‘exercise snacking’ sessions. The exercise intervention was rated as enjoyable (75% reported their enjoyment as ≥4 on a 5-point Likert scale), easy to perform, and most (82%) planned to continue similar exercise at home. We also found it was feasible to assess measures of physical function via videoconferencing, although effect sizes for 4-week changes in both 5-STS (*d* range, 0.4–1.4) and 30-STS (*d* range, 0.7–0.9) following the exercise intervention were similar to controls (*d* = 1.1 and 1.0 for 5-STS and 30-STS, respectively).

**Conclusions:**

Resistance ‘exercise snacking’ may be a feasible strategy for engaging older adults in home-based resistance exercise when delivered and monitored remotely. The findings of this pilot feasibility trial support the need for longer-term studies in larger cohorts to determine the effectiveness of resistance ‘exercise snacking’ approaches for improving physical function in older adults.

**Trial registration:**

The trial was retrospectively registered on 10/11/2021 with the Australian New Zealand Clinical Trials Registry (ANZCTR) (ACTRN12621001538831).

## Background

Age-related declines in skeletal muscle mass, strength, power, and functional capacity strongly influence morbidity, mortality, and quality-of-life in later life [1]. Resistance exercise is the most effective countermeasure for age-related neuromuscular impairments. Despite its wide-ranging benefits, only 6% of adults aged 50 years and older meet current resistance (muscle strengthening) exercise guidelines [2]. This highlights the need to identify more feasible and effective resistance exercise modalities, particularly for older adults. The reason(s) for poor engagement of older adults with resistance exercise are multifactorial, but include a high perceived difficulty, fear of injury, time constraints, and lack of interest or knowledge [3–5].

There are several barriers to participation in supervised, community-based exercise programs in older adults, including time constraints associated with travel to an exercise facility [6], lack of transport [5, 7], cost [8], and a dislike of exercise facilities and group activities [8]. In addition, physical distancing measures associated with the COVID-19 pandemic have also limited engagement in community-based exercise programs, while also presenting challenges for research studies involving supervised in-person exercise sessions and/or physical assessments [9]. There is a need, therefore, to identify feasible and safe exercise interventions that can be performed remotely by older adults, and to determine the feasibility of remote physical function assessments required to evaluate the effectiveness of such interventions.

There has been increased interest in pragmatic and time-efficient exercise modalities (e.g., bodyweight exercise, stair climbing) as potentially more feasible approaches to improving health and fitness [10, 11]. Such ‘minimal-dose’ exercise approaches are supported by findings that regular short bouts (even 5 minutes) of moderate-to-vigorous physical activity (MVPA) are associated with decreased mortality [12]. To date, most studies of pragmatic exercise strategies (sometimes termed ‘exercise snacking’) have focused on exercise for improving cardiorespiratory fitness and/or markers of metabolic health [13–16] rather than on enhancing muscle mass, strength, or function [17]. In one study, ten-minute resistance ‘exercise snacks’ improved 60-second sit-to-stand performance (+ 31%, with no change for control) when performed unsupervised twice daily for 28 consecutive days in older adults (mean age 70 y) [17]. Whether there are potential dose-response effects on the feasibility and safety of similar resistance ‘exercise snacking’ approaches in older adults, as well as their acceptability in this population, remains unclear.

This 4-week pilot randomised controlled trial aimed to determine, in community-dwelling older adults, the feasibility of undertaking a home-based resistance ‘exercise snacking’ intervention (performed either once, twice, or thrice daily) when delivered and monitored remotely. Secondary aims were to determine, in this population, the: 1) feasibility of performing home-based physical function assessments remotely using videoconferencing, 2) effects of the resistance ‘exercise snacking’ intervention on physical function, and 3) acceptability of the resistance ‘exercise snacking’ intervention when performed remotely within the home environment.

## Methods

### Study design and setting

This study was a four-arm, pre-post, 4-week pilot feasibility randomised controlled trial that was designed to determine, in community-dwelling older adults, the feasibility and acceptability of undertaking a home-based resistance ‘exercise snacking’ intervention (performed either once, twice, or thrice daily). The study was conducted during November to December 2020 in Melbourne, Australia. The study period occurred shortly after an extended period of lockdown restrictions (including stay at home orders) as a result of the COVID-19 pandemic, which were in place in Melbourne, Australia between July 8th and October 28th, 2020. All study procedures, including the recruitment and screening of participants, completion of physical function assessments, and the delivery and monitoring of the exercise intervention, were performed remotely without any in-person contact between participants and the research team. Ethics approval was obtained from the Deakin University Human Research Ethics Committee (HREC 2020–011) and all participants provided written informed consent. The trial was retrospectively registered on 10/11/2021 with the Australian New Zealand Clinical Trials Registry (ANZCTR) (ACTRN12621001538831).

### Participants and recruitment

A total of 38 community-dwelling older adults (convenience sample) aged 65–80 years were recruited (during November 2020) via email addresses listed in a database of participants from previous exercise intervention trials conducted within the Institute for Physical Activity and Nutrition (IPAN) who provided consent to be re-contacted for future trials. Participants were deemed eligible to participate if they were: 1) English-speaking, 2) non-smoking, 3) able to walk unaided or with minimal assistance for ≥50 m, 4) cognitively intact as indicated by a score of ≤2 on the Short Portable Mental Status Questionnaire (SPMSQ), and 5) had access to a computer, smart phone or tablet device with a stable network or internet/WiFi connection. Participants were excluded based on the following criteria: 1) participating in structured resistance training more than once per week in the previous 3 months, 2) acute or terminal illness likely to impact study involvement, 3) unstable or ongoing cardiovascular, metabolic, or respiratory disorders, 4) current use of insulin or corticosteroids that could influence skeletal muscle metabolism, 5) self-reported body mass index (BMI) ≥40 kg·m^− 2^, 6) musculoskeletal or neurological disorders impacting voluntary movement, or 7) inability to commit to the study and its requirements. The risk of participants experiencing an adverse event during exercise was determined using the Exercise and Sports Science Australia (ESSA) Adult Pre-exercise Screening System (APSS) [18]. Participants with signs or symptoms of unstable or unmanaged disease (i.e., if participants answered ‘yes’ to any of the Stage 1 questions of the ESSA APSS) were excluded. All participant screening procedures were completed over the telephone, which included gathering responses to the SPMSQ and APSS questionnaires.

**Fig. 1:**
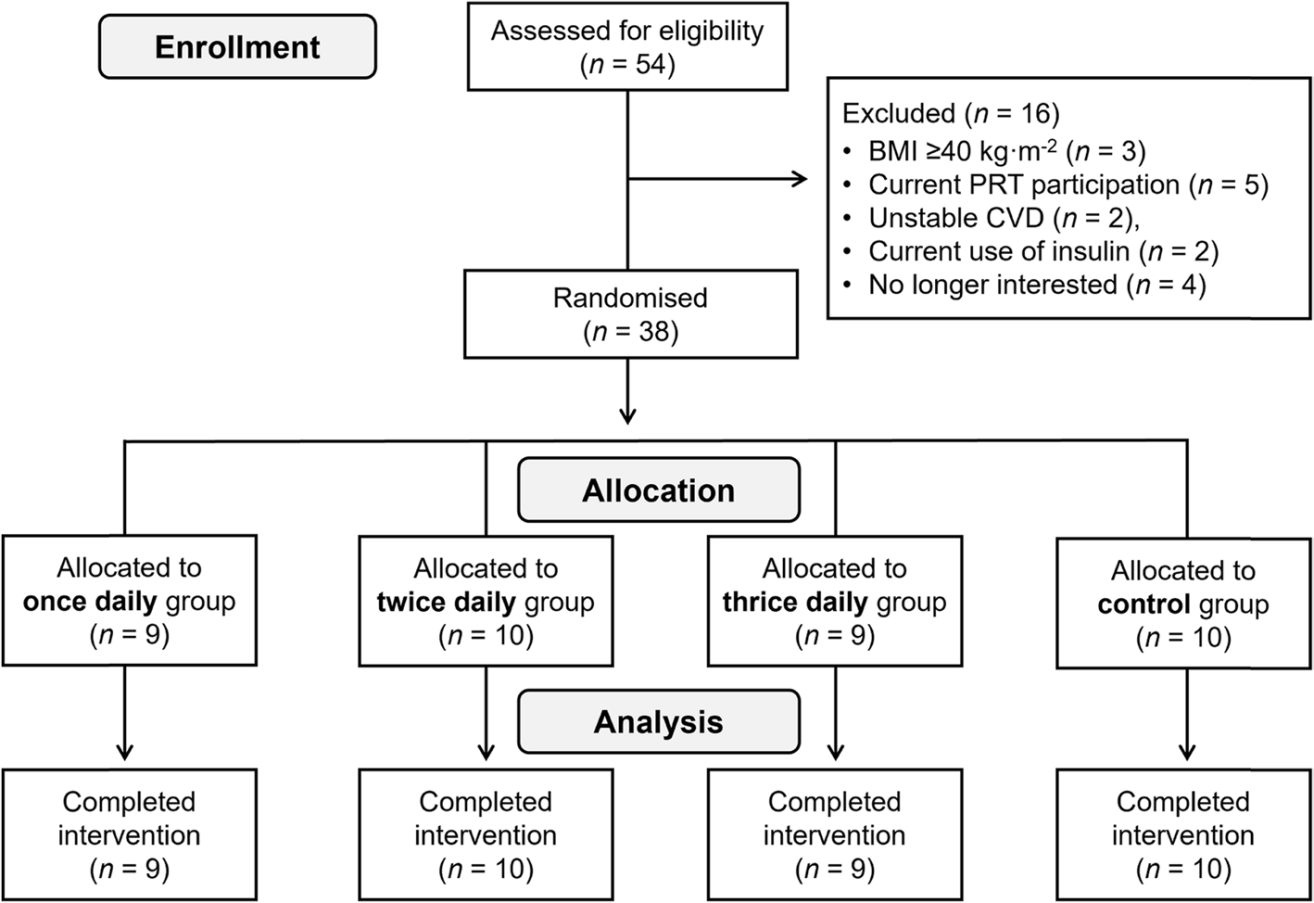
CONSORT diagram

A total of 54 older adults were screened for the study, of which 38 were included. Reasons for exclusion (*n* = 16) are shown in Fig. 1. After screening for eligibility and baseline testing, participants were randomised, stratified by gender, to one of three exercise groups (once-daily group, *n* = 9; twice-daily group, *n* = 10; thrice-daily group, *n* = 9), or a usual-activity control (*n* = 10). Group randomisation was computer-generated (using Microsoft Excel) by an independent person not directly involved in the study.

### Exercise intervention

The exercise intervention involved home-based resistance ‘exercise snacking’ sessions performed either once, twice, or thrice daily for 4 weeks. The exercise program was designed to be pragmatic and time-efficient by: a) focusing on multi-joint exercises involving larger muscle groups; b) targeting improvements in strength (and elements of balance) in lower body muscles most susceptible to age-related declines in muscle mass and strength [19] c) including exercises requiring minimal equipment (i.e., no more than a chair or a step) and no external loading (i.e., bodyweight only), and d) using a time-based prescription that did not require participants to monitor repetitions or sets and allowed the level of effort during each set to be self-regulated.

Each resistance ‘exercise snacking’ session consisted of five exercises that were each performed continuously for 1 min, with 1 min of passive recovery between exercises (total time commitment of 9 min per session). For weeks 1–2, the prescribed exercises were: 1) chair sit-to-stand (no arms), 2) single-leg quarter squat (with chair support), 3) side lunges, 4) calf raise (with chair support), and 5) clock stepping (with chair support). For the clock stepping exercise, participants were instructed to imagine they are standing in the middle of a clock face, and while standing on one leg, to step the other leg forward to the 12 o’clock position and then back to the centre, before repeating this to either the 3 o’clock or 9 o’clock position (for stepping with the right or left leg, respectively) followed by the 6 o’clock position. To provide variety and progressive overload, the five exercises prescribed for weeks 3–4 were changed to: 1) squat into high knee march (knee to elbow), 2) single-leg quarter squat, 3) rapid step ups (involving a rapid concentric phase and performed using a staircase step, or another available step of a similar height), 4) single-leg calf raise, and 5) rapid clock stepping. For unilateral exercises (apart from the squat into high knee march and rapid step ups, both of which were performed in an alternating pattern), one leg was exercised for the first 30 seconds of the one-minute period, before switching to the alternate leg for the final 30 seconds. Participants were instructed to perform as many repetitions as possible in 1 min with appropriate technique for each exercise, and were encouraged to gradually increase both the number and speed of repetitions performed (with appropriate technique) during the intervention. Participants were not asked to record the number of repetitions performed for each exercise during each session, or to perform each ‘exercise snack’ at specific times during the day – rather they were asked to perform each session when convenient and to distribute multiple sessions (where relevant) throughout the day as much as possible.

The exercise intervention was remotely delivered via a commercially available, web-based exercise programming application (PhysiTrack™) and accompanying end-user application (PhysiApp™) accessible via a computer, smart phone, or tablet. The PhysiTrack™ application was used only to deliver instructions regarding the exercise intervention (including written instructions and video-based demonstrations for each exercise) to participants, and not for monitoring purposes. Rather, immediately after completing each resistance ‘exercise snacking’ session, participants recorded in an exercise diary (designed in Microsoft Word) whether they successfully completed the session (yes or no), their RPE (Rating of Perceived Exertion) using the CR-10 scale [20], and whether any adverse events or incidents were experienced. After the first week of the intervention, participants were asked to return the completed exercise diary to the research team via email at the beginning of each subsequent week.

Participants attended two (one each at baseline and follow-up) live videoconference meetings (conducted via Zoom) with the same member of the research team. At the first meeting, participants completed all baseline assessments, were shown how to access the exercise program and video demonstrations using PhysiApp™, and how to complete the exercise diary (including explanation of the RPE CR-10 scale). The researcher also demonstrated each exercise and ensured participants could perform each exercise with appropriate technique. Participants also received a weekly email from the research team reminding them to return their completed exercise diary for the previous week. In the week following completion of the intervention, a second live videoconference meeting was held during which follow-up assessments were performed.

### Feasibility of the exercise intervention

Feasibility of the exercise intervention was considered based on participant retention within the study and adherence to the intervention. Participant retention was recorded as the number (proportion) of participants who were randomised and completed both the four-week exercise intervention (or control period) and follow-up assessments. Adherence to the exercise intervention was considered as the number of sessions completed as a proportion of the number of planned (prescribed) sessions. In addition, exercise adherence was also reported as the prescribed versus actual (completed): i) number of days exercised per week, ii) total number (frequency) of ‘exercise snacks’ per week, and iii) total number of ‘exercise snacks’ during the four-week intervention. The exercise intervention was considered feasible if participant retention was at least 90%, and if participants completed a mean of 80% of prescribed sessions.

### Feasibility of remote physical function assessments

We also explored whether it was feasible for participants to perform home-based physical function assessments remotely using videoconferencing. This was assessed as the proportion of participants who could successfully complete all physical function assessments during both the baseline and follow-up videoconferencing meetings. In addition, the feasibility of the remote home-based physical function assessments was considered based on the occurrence of barriers regarding equipment availability [e.g., lack of a suitable chair for sit-to-stand (STS) assessments], technical issues (e.g., unstable internet connection), and the availability of sufficient space within the home environment to complete each test and achieve the camera angles necessary for test scoring (e.g., view of the participant’s feet and/or eyes for balance testing).

### Adverse events and incidents

An adverse event was defined as an intervention-related event resulting in absence from, or modification to, the exercise intervention. An adverse incident was defined as a minor intervention-related event (such as muscle or joint soreness/stiffness) not requiring absence from, or modification to, the exercise intervention. Any reported adverse events or incidents were followed up by research staff who contacted participants via phone to obtain further information and to advise whether participants should continue (with any modifications as required) or cease the exercise program and seek medical advice.

### Anthropometry and physical function assessments

Participants self-reported their height and body mass to the nearest 1 cm and 1 kg, respectively. Physical function was assessed using a standing balance testing battery, the five-times STS (5-STS) test, and the 30-second STS (30-STS) test. For all physical function tests, participants wore comfortable shoes or were barefoot, which was noted during baseline assessments and repeated at follow-up. The time-of-day at which physical function testing was performed was not controlled, but testing was conducted at a similar time-of-day at baseline and follow-up for each participant where possible.

Participants ability to maintain balance in three different stance positions (side-by-side, semi-tandem, and tandem) was assessed in accordance with the Short Physical Performance Battery (SPPB) protocol [21]. The ability to maintain balance in the tandem stance position with the eyes closed was also assessed. Participants were asked to stand with their feet in full view of the camera (either on a smartphone, tablet, or webcam) and position themselves close to a wall or bench to provide support if needed. After assuming the correct stance position, participants were instructed they may use their arms, bend their knees, or move their body to maintain their balance, but to not move their feet. Participants scored 1 point for each test in which balance was maintained for 10 seconds, and zero if balance was not maintained (for a maximum total score of 4 points). Where balance could not be maintained for a given test, participants did not perform any further balance tests.

For the 5-STS and 30-STS tests, participants were asked to use a chair that: a) had a firm seat and backrest, b) had no arm rests or wheels, and c) was at a height such that participants could place their feet flat on the floor while their upper body was in contact with the backrest. The same chair was used by each participant for both baseline and follow-up assessments. Before commencement of STS testing, participants were asked to position their chair side-on to the camera, and to adjust their camera so that their entire body was visible to the researcher when seated on the chair. If space limitations did not permit their entire body being visible on camera, the position of the chair and/or camera were adjusted so that at a minimum, the seat and backrest were within camera view.

For the 5-STS test, participants began from a seated position in the chair, with their arms folded across the chest, and were instructed to stand fully upright and then return to the seated position five times as quickly as possible. The final score was recorded as the time taken to perform five STS repetitions from initially leaving the chair to being seated after the fifth repetition. The 30-STS test was then performed after a 5-minute recovery following completion of the 5-STS. For the 30-STS test, participants performed repeated chair stands in a manner identical to the 5-STS test; however, they were instructed to instead complete as many repetitions as possible in 30 seconds. The final score was recorded as the number of complete sit-to-stands (defined as standing in a fully upright position) achieved in 30 seconds. Participants were unable to view the 30-second timer during the test and were not provided with any feedback other than when to start and stop the test.

### Acceptability of the exercise intervention

Upon trial completion, process measures were collected (via Qualtrics software) using an author-derived questionnaire completed by participants to evaluate their experiences with, and perceptions about, the exercise intervention. Participants were asked to rate their level of enjoyment of the resistance ‘exercise snacking’ program on a 5-point Likert scale (1 = ‘not at all’, 2 = ‘a little’, 3 = ‘a moderate amount’, 4 = ‘a lot’, and 5 = ‘a great deal’), and if they planned to continue undertaking some form of resistance ‘exercise snacking’ exercise at home. Participants were also asked open-ended questions about what they liked and disliked about the resistance ‘exercise snacking’ program. In addition, if participants indicated they did not plan to continue performing similar exercises after completion of the intervention, they were asked to explain why this was the case, and to describe anything that could be modified about the exercise program to improve the likelihood they would continue performing a similar program at home. All open-ended questions were analysed (within the intervention groups) by researcher(s) (JF) in Microsoft Excel using a general inductive thematic approach [22].

### Statistical analysis

As this was a pilot feasibility study [23], a convenience sample of 38 older adults was recruited and no sample size calculations were performed [24]. All baseline and follow-up data are presented as means ± SDs and all change data are reported as means with 95% confidence intervals (CIs) unless otherwise stated. Data relating to the feasibility of the intervention (i.e., participant retention and intervention adherence) was considered as descriptive in nature. Exploratory analysis was undertaken to evaluate within-group changes in 5-STS and 30-STS test performance using paired samples *t-*tests. Effect sizes (Cohen’s *d*) for within-group changes between baseline and week 4 were calculated according to the following formula: mean follow-up score minus mean baseline score divided by baseline standard deviation, and interpreted as < 0.2 = trivial, 0.2 to < 0.5 = small, 0.5 to < 0.8 = moderate, and ≥ 0.8 = large [25]. Mean changes, ES values, and associated 95% CIs were calculated using JASP software (Version 0.14.1, JASP Team, The Netherlands).

## Results

### Baseline characteristics

A summary of participant characteristics for each intervention and control group at baseline is shown in Table 1.

**Table 1:** Baseline participant characteristics

	Resistance ‘Exercise Snacking’ Dose			
	Once-daily	Twice-daily	Thrice-daily	Control
***n***	9	10	9	10
Age (years)	69.9 ± 5.3	68.9 ± 2.9	69.8 ± 3.0	69.8 ± 3.5
Sex (% female), *n* (%)	6 (67)	6 (60)	6 (67)	6 (60)
Height (cm)	166.6 ± 8.0	168.4 ± 10.4	165.0 ± 8.1	164.2 ± 8.2
Weight (kg)	73.6 ± 11.5	78.5 ± 15.4	76.1 ± 12.3	76.9 ± 16.7
BMI (kg·m^−2^)	25.9 ± 3.8	27.1 ± 5.7	27.8 ± 5.8	27.9 ± 4.2
Overweight *n* (%)	3 (33)	4 (40)	4 (44)	7 (70)
Obese, *n* (%)	2 (22)	2 (20)	2 (22)	2 (20)
Hypertension (treated), *n* (%)	2 (22)	6 (60)	6 (67)	5 (50)
Type 2 diabetes, *n* (%)	4 (44)	4 (40)	1 (11)	2 (20)
Musculoskeletal or neurological conditions, *n* (%)	0 (0)	2 (20)	1 (11)	4 (40)
Osteoporosis, *n*	0	1	0	1
Osteoarthritis (knee), *n*	0	0	0	2
Previous hip replacement surgery, *n*	0	1	0	0
Previous knee replacement surgery, *n*	0	0	0	1
Previous rotator cuff surgery, *n*	0	0	0	1
Musculoskeletal (gluteal) injury, *n*	0	0	1	0
Spinal complaints, *n*	0	0	0	1

Values are mean ± SD or number of participants (percentage); Body mass index (BMI); overweight BMI 25–29.9 kg·m^−2^; obese BMI ≥30 kg·m^− 2^

### Feasibility of the exercise intervention

As shown in Fig. 1, participant retention was 100% in all intervention groups and controls. Mean adherence to the exercise intervention for all exercise groups combined was 87% ± 20%, which was similar between the three exercise groups [mean (95% CI): once-daily, 97% (95, 100); twice-daily, 82% (63, 101); thrice-daily, 81% (68, 94)]. Additional adherence data summarising the prescribed versus actual (completed) for i) number of days exercised per week, ii) number (frequency) of ‘exercise snacks’ sessions per week, and iii) total number of ‘exercise snacks’ over the 4-week intervention is shown in Table 2.

**Table 2:** Adherence to the resistance ‘exercise snacking’ intervention presented as the prescribed versus actual (completed) for: i) number of days exercised per week, ii) number (frequency) of ‘exercise snacks’ per week, and iii) total number of ‘exercise snacks’ (both per participant and for all participants combined) during the four-week intervention

	Days exercised per week		Number (frequency) of ‘exercise snack’ sessions per week		Total number of ‘exercise snacks’ over 4 weeks			
					Per participant		All participants	
Group	Prescribed	Actual Mean (SD)	Prescribed	Actual Mean (SD)	Prescribed	Actual Mean (SD)	Prescribed	Actual
Once-daily	7	7 (0)	7	7 (0)	28	27 (1)	252	245
Twice-daily	7	6 (2)	14	11 (4)	56	46 (15)	560	459
Thrice-daily	7	6 (1)	21	17 (4)	84	68 (14)	756	613

### Feasibility of remote physical function assessments

All 38 participants were able to successfully complete all physical function assessments (balance testing battery, 5-STS, and 30-STS) within their homes during the live videoconference meetings with the research staff at both baseline and follow-up.

### Adverse events and incidents

Two participants (2/28 or 7%) allocated to the exercise groups each reported a single adverse event during the intervention, which included one occurrence of plantar fasciitis and one occurrence of lower back and leg pain associated with a spinal nerve/disc injury. In both cases, these reported adverse events were sufficiently minor to allow participants to continue the intervention with some minor exercise modifications (e.g., limiting range-of-motion and/or completing fewer repetitions in certain exercises, or avoiding unilateral exercises on the affected side) as required. Seven participants (7/28 or 25%) allocated to the exercise groups reported a total of eight minor adverse incidents during the intervention, each of which involved minor musculoskeletal complaints such as muscle (thigh or calf) or joint (knee or ankle) stiffness and/or soreness that did not affect their participation.

### Rating of perceived exertion (RPE) responses to exercise

Average (95% CI) RPE (CR-10 scale) during the intervention was similar between the once-daily [4.0 (3.2. 4.8)], twice-daily [2.7 (1,7, 3.7)], and thrice-daily [3.4 (2.7, 4.1)] groups.

### Physical function outcomes

With the exception of the once daily exercise group, effect sizes for improvements in 5-STS time after 4 weeks were similar (*d* range, 1.1–1.4) for the twice and thrice daily exercise groups and controls (Table 3). Effect sizes for changes in 30-s STS performance after 4-weeks were also similar (*d* range, 0.7–1.0) for all exercise groups and controls (Table 3). For balance, 50 to 78% of participants within each group reported no changes after 4 weeks (Table 4).

**Table 3:** Baseline and 4-week results for the 5 times sit-to-stand (5-STS) and 30-second STS test and the within-group changes relative to baseline and effect sizes (Cohen’s *d*) for each of the groups

	Baseline (***n*** = 38)	4-weeks (***n*** = 38)	Mean change (95% CI)	Cohen’s ***d*** effect size (95% CI)
**5-STS (s)**				
Once daily	12.9 (2.4)	11.9 (3.3)	−1.0 (−3.0, 1.0)	−0.4 (−1.1, 0.3)
Twice daily	14.0 (2.6)	12.2 (2.2)	−1.8 (−2.8, −0.8)	−1.4 (−2.1, −0.4)
Thrice daily	14.9 (2.6)	12.3 (2.5)	− 2.6 (−4.4, − 0.8)	−1.1 (−2.0, −0.3)
Control	14.7 (3.1)	13.1 (3.1)	− 1.7 (−2.8, −0.6)	−1.1 (−1.9, −0.3)
**30-STS (number of stands)**				
Once daily	13.1 (2.3)	14.3 (3.2)	1.2 (−0.2,2.6)	0.7 (−01, 1.4)
Twice daily	11.7 (2.1)	13.1 (2.2)	1.4 (0.2, 2.6)	0.8 (0.1, 1.5)
Thrice daily	11.4 (1.5)	12.7 (2.1)	1.2 (0.2, 2.2)	0.9 (0.1, 1.7)
Control	11.9 (2.6)	12.7 (2.8)	0.8 (0.2, 1.4)	1.0 (0.2, 1.8)

Baseline and 4-week values are means (standard deviations)

**Table 4:** Baseline and follow-up scores for the balance test and the number and proportion of participants that experienced an increase, no change, or a decrease in their balance score after 4 weeks

Group	Baseline (***n*** = 38)	4-weeks (***n*** = 38)	Increase	No change	Decrease
Once daily	3 (3, 4)	4 (3, 4)	3 (33%)	5 (56%)	1 (11%)
Twice daily	3 (3, 4)	3 (3, 4)	2 (20%)	5 (50%)	3 (30%)
Thrice daily	4 (3, 4)	3 (3, 4)	0 (0%)	7 (78%)	2 (22%)
Control	4 (3, 4)	3 (3, 4)	1 (12%)	5 (63%)	2 (25%)

Baseline and 4-week values are median (interquartile range)

### Acceptability of the intervention

Seventy-five percent (21/28) of participants allocated to the exercise groups rated their level of enjoyment to the exercise program as either “a great deal” or “a lot” (39%, “a great deal”; 36%, “a lot”; 18%, “a moderate amount”; 4%, “a little”; 0%, “not at all”). Seventy-nine percent (22/28) of participants indicated they would continue performing similar resistance ‘exercise snacking’ at home following the intervention. With regards to acceptability of the intervention, a number of key themes were identified and are summarised (with representative quotes) in Table 5. Participants favoured the brief and frequent nature of the exercise sessions, which allowed integration of the exercise session(s) into their day without interfering with their daily routine. Participants also noted the exercises were relatively easy (particularly in the first 2 weeks of the program) and required minimal equipment to perform, both of which improved the feasibility of the program and had positive implications for improved exercise self-efficacy. There were also indications participants appreciated that the program, despite being brief, addressed important aspects of their health. The responses from some participants suggested future interventions would benefit from individualising exercises selection to suit both technical competency and personal preferences. For example, some participants found the exercise progression between weeks 1–2 and weeks 3–4 of the intervention to be too challenging, while others noted they would have preferred additional exercise variety, the inclusion of upper-body exercises, and incorporating external loads (weights) into the exercises.

**Table 5:** Examples of key response themes and example quotes from participants related to the acceptability of the intervention

Response theme	Example participant responses
**The exercise program could easily be integrated into the daily routine of participants**	“(The exercise program) didn’t interfere with my daily routine. So it was easy to adhere to the program.” ***Male, aged 77 years (allocated to thrice-daily exercise group).***
	“(I liked) that I can add it to my routine of exercises during the day.” ***Male, aged 72 years (allocated to thrice-daily exercise group).***
**The exercise program was feasible as it was relatively quick, easy, and required minimal equipment**	“(The exercise program) forced me to set some time aside each day to exercise - otherwise, I tend to put an exercise regimen on the back burner each day, telling myself ‘I’ll do it later’.” ***Female, aged 66 years (allocated to once-daily exercise group).***
	“I liked the fact that the exercises were pretty easy and dispensed with quickly and were spaced out over the day. They were very easy to do and didn’t require setting up any equipment, etc.” ***Female, aged 66 years (allocated to twice-daily exercise group).***
**The exercise program addressed important aspects of health**	“I liked that (the exercise program) was short but worked on important things (such as) balance, muscle strength, agility.” ***Female, aged 75 years (allocated to twice-daily exercise group).***
	“The regular exercise has improved my strength and flexibility somewhat” ***Male, aged 77 years (allocated to once-daily exercise group).*** “(The exercise program) also reinforced for me the need to do more of these types of exercises to try to improve my balance and muscle tone.” ***Female, aged 66 years (allocated to once-daily exercise group).***
**The exercise program promoted greater exercise engagement and self-efficacy**	“(I liked) learning how much exercise I could do, as I usually only walk on a daily basis.” ***Female, aged 67 years (allocated to once-daily exercise group).***
	“Covid (COVID-19 pandemic) resulted in a lack of some exercise for me and this programme forced me to make a greater effort than I had been. I ‘feel’ fitter as a result. Thanks for the experience.” ***Male, aged 70 years (allocated to twice-daily exercise group).***
	“I know that I should do more physical activity so it was good that I had to do the exercises twice a day. My family were pleased also!” ***Female, aged 67 years (allocated to twice-daily exercise group).***
**Exercise selection and progression may need to be individualised for some participants**	“This is a good series. I found the step up between part 1 and part 2 a little extreme and cannot fully comply with the elbow to knee exercise at this time. I feel the exercises and the timing were just about right to encourage a senior to resume exercise.” ***Male, aged 77 years (allocated to once-daily exercise group).*** “(The) gradient between week 1–2 and week 3–4 exercises was a bit too extreme.” ***Male, aged 68 years (allocated to once-daily exercise group).*** “(The) exercises (were) easy and appropriate for the age group in the study”. ***Female, aged 70 years (allocated to twice-daily exercise group).***
**The exercise program may be improved with additional exercise variety, the inclusion of external loads, and upper-body exercises**	“I’d like to incorporate some more diverse exercises in the program, such as lifting weights or some such (sic). I really feel the need to tone up my arms in particular.” ***Female, aged 66 years (allocated to once-daily exercise group).*** “The variety was good. (All) in all, there were about 8 different exercises. Further variety would be good, but I’ll source some alternative exercises myself. I’ll also see if I can convince any of my friends (all “older” people) to try them, but I don’t expect much luck there!” ***Male, aged 67 years (allocated to once-daily exercise group).*** “I thought it was good. Maybe some more upper body strength exercises (would improve the program).” ***Female, aged 75 years (allocated to twice-daily exercise group).*** “(The) use of weights and other balancing actions (would improve the program).” ***Female, aged 74 years (allocated to once-daily exercise group).***

## Discussion

The findings of this pilot randomised controlled trial suggest a pragmatic resistance ‘exercise snacking’ intervention is feasible when performed by community-dwelling older adults in their homes either once, twice, or thrice daily for 4 weeks, and when delivered and monitored entirely remotely. The feasibility of the exercise intervention was evidenced by the 100% participant retention and high adherence to the intervention (87% overall). The intervention was also safe, with only two adverse events occurring from a total of 1317 completed resistance ‘exercise snacking’ sessions over the 4-week period. All participants successfully completed all remote physical function assessments at both baseline and follow-up, suggesting it was feasible for older adults to perform these assessments within their home environment using videoconferencing. Exploratory analysis suggested there was little evidence the intervention was effective for improving measures of physical function, although these findings should be considered in context of the short-term nature of this trial and the small convenience sample size. Finally, participants overall found undertaking the home-based exercise intervention to be acceptable, with most (82%) indicating they would continue performing similar exercise at home after completing the intervention.

Consistent with our findings regarding the feasibility and safety of home-based and unsupervised resistance ‘exercise snacking’, a previous study in which older adults underwent a similar home-based and unsupervised exercise protocol twice-daily for 4 weeks reported 98% adherence to the intervention and no adverse events [17]. This adherence rate was higher than the mean adherence (87%) for all exercise frequency groups in the present study, which may be attributed to the higher frequencies used in the thrice daily group, in which mean adherence was 81% (although between-group differences in adherence were not statistically significant). The present findings nevertheless highlight it is possible for older adults to adhere to either once, twice or thrice daily bouts of exercise for 4 weeks. In addition, we found it was feasible for community-dwelling older adults to complete physical function assessments remotely within their homes using videoconferencing. All 38 participants in this study successfully completed all physical function assessments remotely at both baseline and follow-up, and there were no reported issues with potential barriers such as the availability of equipment (e.g., appropriate chair for STS testing), having sufficient physical space within the home environment to perform the tests, or technical issues with the videoconferencing technology (i.e., Zoom). Although further validation work is needed in larger populations of older adults, these findings are supported by recent observations [9] that various physical function assessments (including the 5-STS and 30-STS tests) are both valid and reliable (ICC > 0.70) when conducted remotely compared to when performed face-to-face. Together these findings have promising implications for monitoring the effectiveness of remotely delivered exercise programs aimed at improving physical function in older adults.

The positive findings regarding the feasibility of the intervention should also be considered in context of the manner in which the intervention was monitored remotely, which has potential implications for the broader feasibility and scalability of this pragmatic exercise approach. While the PhysiTrack™ application (and associated end-user application PhysiApp™) was used to deliver instructions regarding the exercise intervention to participants, intervention adherence, RPE, or adverse events were instead monitored using a custom exercise diary. This monitoring was also undertaken relatively infrequently (i.e., only once per week), and researchers did not receive any real-time information or notifications on factors related to the monitoring of the intervention. Given the pragmatic nature of the exercises within the intervention, we believe it is possible that older adults could successfully perform these exercises in the absence of video-based demonstrations (as were provided via PhysiApp™). The exercise program may therefore be feasible for individuals or clinicians who are without access to similar exercise prescription software. Although the intervention was monitored asynchronously and relatively infrequently, it is possible that removing or reducing the frequency of monitoring could have negatively influenced adherence to the intervention. However, considering both the safety of the intervention and its pragmatic nature, it is possible that exercise adherence may have remained high even if monitoring was less frequent or absent, given that participants successfully completed the program unsupervised and without receiving any feedback during the program (e.g., on exercise technique or progression). Taken together, the simple and infrequent manner in which the intervention was delivered and monitored (and the associated low time burden for health professionals) has positive implications for the broader feasibility of similar remote exercise interventions in older adults and their potential to be rolled out at scale. While the initial evidence seems positive, future implementation trials are required to determine the feasibility of similar remote exercise interventions in clinical settings when administered by health professionals.

The qualitative analyses in this study revealed important insights regarding the acceptability of the resistance ‘exercise snacking’ program to participants, which may have implications for the long-term feasibility of this novel exercise approach. There were indications that the pragmatic characteristics of the exercise intervention, including that it was time-efficient and incorporated exercises that were simple and required minimal equipment, were particularly appreciated by participants. These factors allowed participants to feasibly integrate the exercise sessions into their day, even when the program was performed on multiple occasions (i.e., up to three times daily). This is supported by the observation that adherence to the program remained high for all daily exercise frequencies, although mean adherence was 16–17% lower in those assigned to twice- or thrice-daily exercise compared to once-daily exercise snacking sessions. Participants also felt that, despite its pragmatic nature, the exercise program addressed important aspects of their health (e.g., improvements in muscle strength and balance). Further, the exercise program appeared to promote greater self-efficacy with exercise, with some participants suggesting the exercise program highlighted they were capable of more exercise than they previously believed. When asked if anything could be modified about the exercise program to improve the likelihood that they would continue performing similar exercise at home, some responses from participants highlighted the potential need to individualise exercise selection to suit both differences in technical ability and personal preferences. While the exercise modifications introduced in weeks 3–4 of the intervention were aimed at providing additional variety and progressive overload, some participants highlighted they found some of the new exercises difficult to perform. It was also clear that future ‘exercise snacking’ interventions could be further improved by providing additional exercise variety (even if the technical demands remain similar), which may be more prudent given the high-frequency – and therefore potentially repetitive – nature of an ‘exercise snacking’ approach, in addition to the inclusion of upper-body exercises. Some participants also expressed the desire to include external loading (i.e., weights) into the program, suggesting a need for future studies to explore potential solutions that are feasible within the home environment (e.g., use of resistance bands, small portable weights, or household items). Taken together, these insights support the notion that ‘exercise snacking’ is a feasible and acceptable resistance exercise modality when performed remotely by community-dwelling older adults. While our findings in this regard were overall positive, it should be considered that the acceptability of the exercise intervention to participants may have been influenced by, and should be interpreted in the context of, their previous experiences in participating in exercise-related studies. Given COVID-19 restrictions (including ‘stay at home’ orders) had only recently been lifted when the study period began, it also remains possible that participants may have continued to spend additional time within their home environment during the time in which the study was conducted, which may have positively influenced the feasibility and/or acceptability of the home-based program.

While this short-term pilot trial was not designed nor adequately powered to detect between-group differences in measures of physical function, the modest changes in 5-STS and 30-STS performance (but not balance) at follow-up appeared to be similar across all three intervention groups and controls. Although physical function assessments performed remotely via videoconferencing show acceptable validity and reliability when compared to face-to-face assessments [9], the similar changes in 5-STS and 30-STS test performance in both the intervention and control groups may be explained by learning effects, particularly given the lack of test familiarisation, which may have reduced the sensitivity of these measures for detecting changes in physical function over time. While the short-term duration of the exercise intervention also likely influenced the magnitude of any changes (or between group differences) in physical function, previous findings suggest similar pragmatic and unsupervised resistance exercise approaches can be effective for improving measures of physical function (e.g., 60-second sit-to-stand performance) after 4 weeks [17]. Nevertheless, our findings are consistent with another study [26] that also showed no difference in changes in physical function measures assessed remotely using videoconferencing after 4 weeks of twice-daily ‘exercise snacking’ versus controls. Longer-term studies in larger cohorts are therefore needed to confirm these preliminary findings and determine whether similar resistance ‘exercise snacking’ approaches, and potentially those incorporating additional exercise variety, both upper- and lower-body exercises, and external resistance (e.g., via resistance bands and/or small weights), are effective for improving physical function in older adults.

## Limitations

The limitations of this study must be considered when interpreting the findings. As all physical function assessments were conducted remotely, this may have influenced the validity and/or the reliability of these assessments and consequently the changes in physical function observed in this study. Given study participants had previously participated in exercise intervention trials, this suggests they were both a relatively healthy and physically active cohort, which may have influenced the magnitude of physical function changes from baseline. In support of this, only 32% of participants had baseline 5-STS scores greater than 15 seconds, which is a cut-point used to indicate low muscle strength according to the European Working Group on Sarcopenia in Older People (EWGSOP2) consensus guidelines [27]. Given their history of participation in exercise intervention trials, this may have positively influenced the feasibility and/or acceptability of participants undertaking the home-based intervention when delivered and monitored remotely, and the feasibility of performing home-based physical function assessments remotely using videoconferencing. It is therefore unclear whether the remote exercise intervention and/or physical function assessments may be similarly feasible or acceptable in older adults without a similar history of participation in exercise-related studies. Recruitment from this cohort also cannot inform the feasibility of recruitment from the general population for a larger implementation and/or effectiveness trial. Finally, given all study participants were required to have sufficient digital literacy and access to a stable internet/WiFi connection to be included in the study, this may limit the generalisability of the findings to the broader population. Despite these limitations, the positive findings on the adherence to, and acceptability of, the exercise intervention suggests resistance ‘exercise snacking’ may be a promising approach for engaging older adults with resistance-type exercise.

## Conclusions

This pilot randomised controlled trial suggests a remotely delivered and monitored pragmatic resistance ‘exercise snacking’ intervention is acceptable and can be safely and feasibly performed by community-dwelling older adults within their homes either once, twice, or thrice daily for 4 weeks. However, further longer-term studies in larger cohorts are required to determine the effectiveness of home-based pragmatic resistance ‘exercise snacking’ approaches for improving physical function in older adults when delivered and monitored remotely.

## Data Availability

The datasets generated and/or analysed during the current study are not publicly available due to ethical restrictions but are available from the corresponding author on reasonable request.

## Abbreviations

30-STS: 30-second sit-to-stand test
5-STS: Five-times sit-to-stand test
ANZCTR: Australian New Zealand Clinical Trials Registry
APSS: Adult Pre-exercise Screening System
BMI: Body mass index
ESSA: Exercise and Sports Science Australia
IPAN: Institute for Physical Activity and Nutrition
MVPA: Moderate-to-vigorous physical activity
RPE: Rating of Perceived Exertion
SPMSQ: Short Portable Mental Status Questionnaire
SPPB: Short Physical Performance Battery
